# Evaluation of a renal risk score for Japanese patients with ANCA-associated glomerulonephritis in a multi-center cohort study

**DOI:** 10.3389/fimmu.2023.1141407

**Published:** 2023-02-28

**Authors:** Tomohisa Uchida, Kunihiro Ichinose, Ayuko Yamashita, Kumiko Muta, Mineaki Kitamura, Shuntaro Sato, Naoki Iwamoto, Tomoya Nishino, Atsushi Kawakami

**Affiliations:** ^1^ Department of Rheumatology, National Hospital Organization Ureshino Medical Center, Ureshino, Japan; ^2^ Department of Immunology and Rheumatology, Division of Advanced Preventive Medical Sciences, Nagasaki University Graduate School of Biomedical Sciences, Nagasaki, Japan; ^3^ Department of Rheumatology, Shimane University Faculty of Medicine, Izumo, Japan; ^4^ Department of Nephrology, Nagasaki University Hospital, Nagasaki, Japan; ^5^ Clinical Research Center, Nagasaki University Hospital, Nagasaki, Japan

**Keywords:** ANCA, ANCA-associated glomerulonephritis, end-stage renal disease, renal risk score, histological classification

## Abstract

**Background:**

In patients with anti-neutrophil cytoplasmic antibody (ANCA)-associated glomerulonephritis, prediction of renal survival should guide the choice of therapy, but a prediction of the histological classification has inconsistencies.

**Objectives:**

To evaluate the usefulness of renal risk score (RRS) for Japanese patients with ANCA-associated glomerulonephritis (AAGN) and compare the prediction for end-stage renal disease (ESRD) between RRS and the histological classification.

**Methods:**

We retrospectively analyzed 96 patients with AAGN who underwent a renal biopsy. Renal survival was categorized by RRS, and the histological classification was assessed separately. We compared the predictive values for RRS and the histological classification.

**Results:**

The median observational period was 37.5 (interquartile range [IQR] 21.5–77.0) months. The median RRS point at the time of renal biopsy was 2 (IQR 0–7.8), and the patients were categorized into low- (n = 29), medium- (n = 43), and high-risk groups (n = 24) using RRS. As expected, the renal prognosis was the worst in the “high-risk” group and the best in the “low-risk” group. In the histological classification, the survival deteriorated progressively from “focal” (best) to “mixed,” “crescentic,” and “sclerotic” (worst) classes, different from the order in the original proposal for this system. Multivariable Cox regression analysis revealed that RRS was independently associated with ESRD. The difference in prediction for renal survival between RRS and the histological classification was not significant using area under receiver-operating-characteristic curves.

**Conclusion:**

We evaluated the usefulness of RRS in Japanese patients with AAGN and found it a stable predictor of renal survival in such patients.

## Introduction

Anti-neutrophil cytoplasmic antibody (ANCA)-associated vasculitis (AAV) is a systemic vasculitis of small vessels accompanied by ANCAs (1). The major variants of AAV include eosinophilic granulomatosis polyangiitis (EGPA), granulomatosis polyangiitis (GPA), and microscopic polyangiitis (MPA) (2). Renal involvement is frequent in GPA and MPA, and 20-40% of cases develop the end-stage renal disease (ESRD) within 5 years (3–5). Renal involvement is also associated with a worse prognosis than AAV patients without impaired renal function (4, 6, 7). Clinicians need to perform prompt diagnosis and initiation of adequate immunosuppressive therapy to preserve patient and kidney outcomes, but avoidance of adverse events such as treatment-related complications, infection, cardiovascular diseases, and cancer is also a priority (8). Therefore, careful selection of patients who would benefit from intensive immunosuppressive therapy is highly required. Berden et al. proposed a histological classification to predict renal outcomes in patients with ANCA-associated glomerulonephritis (AAGN) in 2010 (9). However, several meta-analyses demonstrated that the histological classification did not accurately predict the renal prognosis of the mixed and crescentic classes (10–13). Recently, Brix et al. proposed another scoring system, a renal risk score (RRS), for predicting the renal prognosis (14). The RRS differs from the histological classification in that it is based on three parameters: percentage of normal glomeruli, tubular atrophy and interstitial fibrosis rate of the kidney, and estimated glomerular filtration rate (eGFR), which are scored according to the severity and classified into three groups with different prognoses. This scoring system has been validated in several studies and one meta-analysis (15–20). We aimed to validate the usefulness of the RRS for Japanese patients over a long-term observation period.

## Patients and methods

### Patients

The patients with ANCA-associated glomerulonephritis (AAGN) who underwent a renal biopsy at Nagasaki University Hospital and its associated hospitals between 1992 and 2019 were enrolled and assessed for eligibility in this study. The inclusion criteria were as follows: ANCA was detected in the sera; renal biopsy revealed necrotizing and/or crescentic glomerulonephritis; and follow-up of patients lasted at least 12 months (including patients who died or ESRD and required renal replacement therapy within 12 months). EGPA cases were not included because the biological and clinical presentation differed from GPA and MPA cases. Patients with secondary vasculitis or comorbid kidney disease were excluded. The baseline characteristics evaluated were the patient’s age, sex, diagnosis, white blood cell count, hemoglobin, C-reactive protein (CRP), eGFR calculated as per the Japanese-based equation: eGFR (ml/min/1.73 m^2^) = 194 × serum creatinine ^−1.094^ × age^−0.287^ (if female, ×0.739) (21), proteinuria, hematuria, ANCA subtype determined by indirect immunofluorescence or enzyme-linked immunosorbent assay, use of antidiabetic drugs, smoking history, hypotensive drug, and immunosuppressive medication. The Birmingham Vasculitis Activity Score (BVAS) was used to assess the activity of the disease at the initial presentation (22). An opt-out strategy was chosen for the informed consent procedure; data from those who indicated an unwillingness to participate were excluded. This study was reviewed and approved by the Institutional Review Boards of Nagasaki University (approval no. 20021012).

### Histopathologic evaluations

Biopsies were independently scored by two expert nephrologists blinded to clinical data (KM, MK). All specimens had at least five glomeruli per biopsy. Various calculations were performed for the RRS assessment, including the percentage of normal glomeruli, tubular atrophy/interstitial fibrosis, and renal function at the time of diagnosis (14). The histological classification was made according to the definition proposed by Berden et al. (9).

### Outcome measures

The primary endpoint was the cumulative percentage of patients who developed ESRD over time censored by death. ESRD was defined as requiring long-term renal replacement therapy or renal transplantation. Renal survival time for each patient was calculated from the time of biopsy to the last time of follow-up or the time point of reaching ESRD.

### Statistical analyses

Data are expressed as a median with an interquartile range (IQR) or n (%). Wilcoxon’s rank sum test was used to compare the continuous variables, and Fisher’s exact test to compare categorical variables. Renal survival was assessed using the Kaplan-Meier method, and differences between survival curves were compared with the log-rank test. Univariate and multivariable Cox proportional hazard regression analyses were performed to identify factors related to ESRD. Variables with p-values <0.15 and factors expected to be associated with the univariate Cox regression analyses were entered in the multivariable Cox regression analyses. Due to the collinearity between the explanatory variables and the RRS scoring system, eGFR was excluded. Discrimination capacity to predict dialysis dependency was assessed using the area under the receiver operating characteristic curve (AUC). We calculated differences using DeLong’s test. P-values <0.05 were considered statistically significant.

Statistical analyses were performed using JMP^®^ Pro 16 software (SAS Institute, Cary, NC) and RStudio version 2022.07.1, an integrated development environment for R version 4.1.2 (R Foundation for Statistical Computing, Vienna, Austria) (23).

## Results

### Baseline characteristics

**Figure 1:**
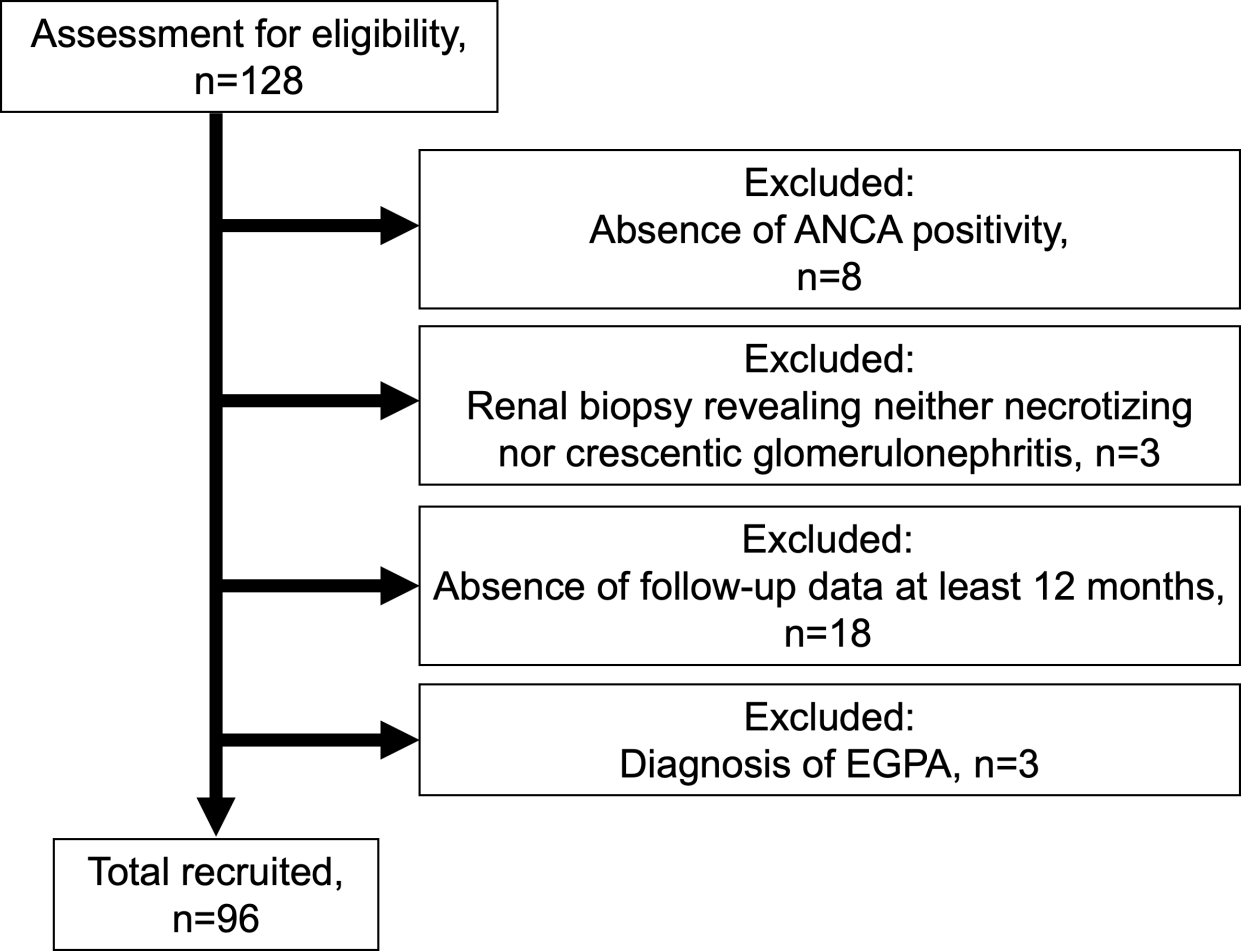
Flowchart summarizing the enrollment of the patient with ANCA-associated renal vasculitis ANCA, anti-neutrophil cytoplasmic antibody; EGPA, eosinophilic granulomatosis polyangiitis.

Of the 128 patients, 96 patients with ANCA-associated renal vasculitis were enrolled (**Figure 1**). The patients included 83 with MPA, 10 with GPA, and 3 with renal-limited vasculitis. The mean BVAS was 14 (IQR 12–18). The baseline characteristics are summarized in **Table 1**, and the comparison of baseline characteristics between the patients who progressed to ESRD or not are summarized in **Supplementary Table S1**.

**Table 1:** Baseline characteristics of patients with ANCA-associated renal vasculitis.

Variables	Total (n = 96)
Male (n, %)	46 (47.9%)
Age, yrs^†^	70 (62.3-76.8)
Diagnosis (n, %)	MPA 83 (86.5%) GPA 10 (10.4%) RLV 3 (3.1%)
WBC (/μL)^†^	8450 (6600-11400)
Hb (g/dL)^†^	9.8 (8.63-11.08)
CRP (mg/dL)^†^	2.7 (0.27-8.86)
eGFR (mL/min/1.73m^2^)^†^	27.6 (15.5-39.6)
Proteinuria (n, %)	95 (99%)
Hematuria (n, %)	97 (100%)
MPO-ANCA positivity (n, %)	92 (95.8%)
PR3-ANCA positivity (n, %)	6 (6.3%)
BVAS^†^	14 (12-18)
Use of hypotensive drugs (n, %)	37 (42.5%) n/a 9 patients
Smoking history (n, %)	35 (43.8%) n/a 16 patients
Use of diabetes mellitus (n, %)	8 (9.0%) n/a 7 patients
Use of Methylprednisolone pulse (%)	61 (63.5%)
Use of glucocorticoid (n, %)	94 (97.9%)
Glucocorticoid dose(mg/day)^†^	40 (30-50)
Plasmapheresis (n, %)	13 (13.5%)
Cyclophosphamide (n, %)	19 (19.8%)
Rituximab (n, %)	2 (2.1%)
ESRD (n, %)	15 (15.6%)

ANCA, anti-neutrophil cytoplasmic antibody; BVAS, Birmingham Vasculitis Activity Score; CRP, C-reactive protein; eGFR, estimated glomerular filtration rate; ESRD, end-stage renal disease; GPA, granulomatosis with polyangiitis; MPA, microscopic polyangiitis; MPO, myeloperoxidase; n/a, not available; PR3, proteinase-3; RLV, renal-limited vasculitis; WBC, white blood cell count

^†^Values are the median with IQR

### Evaluating the patients with RRS and histological classification

The median RRS at diagnosis was 2 (IQR 0–7.8), and the patients were categorized by RRS into low- (n = 29), medium- (n = 43), and high-risk groups (n = 24), respectively. The distribution of the RRS parameters is summarized in **Table 2**. Patients were also classified according to the histological classification (9) as focal (n = 12), crescentic (n = 18), mixed (n = 48), or sclerotic (n = 18). Comparisons of baseline characteristics by RRS and the histological classification are summarized in **Supplementary Tables S2**, **S3**, respectively.

**Table 2:** The distribution of the parameters of renal risk score in patients with ANCA-associated renal vasculitis.

Parameters of renal risk score	No. of patients, n (%)
Percentage of normal glomeruli (N)	
N0 (0 points) >25%	57 (59.4%)
N1 (4 points) 10%-25%	13 (13.5%)
N2 (6 points) <10%	26 (27.1%)
Tubular atrophy/interstitial fibrosis (T)	
T0 (0 points) ≦25%	41 (42.7%)
T1 (2 points) >25%	55 (57.3%)
Renal function at the time of diagnosis (eGFR; G)	
G0 (0 point) >15 mL/min/1.73m^2^	76 (79.2%)
G1 (3 points) ≦15 mL/min/1.73m^2^	20 (20.8%)

ANCA, anti-neutrophil cytoplasmic antibody; eGFR, estimated glomerular filtration rate

### Evaluating renal outcome with RRS and histological classification

**Figure 2:**
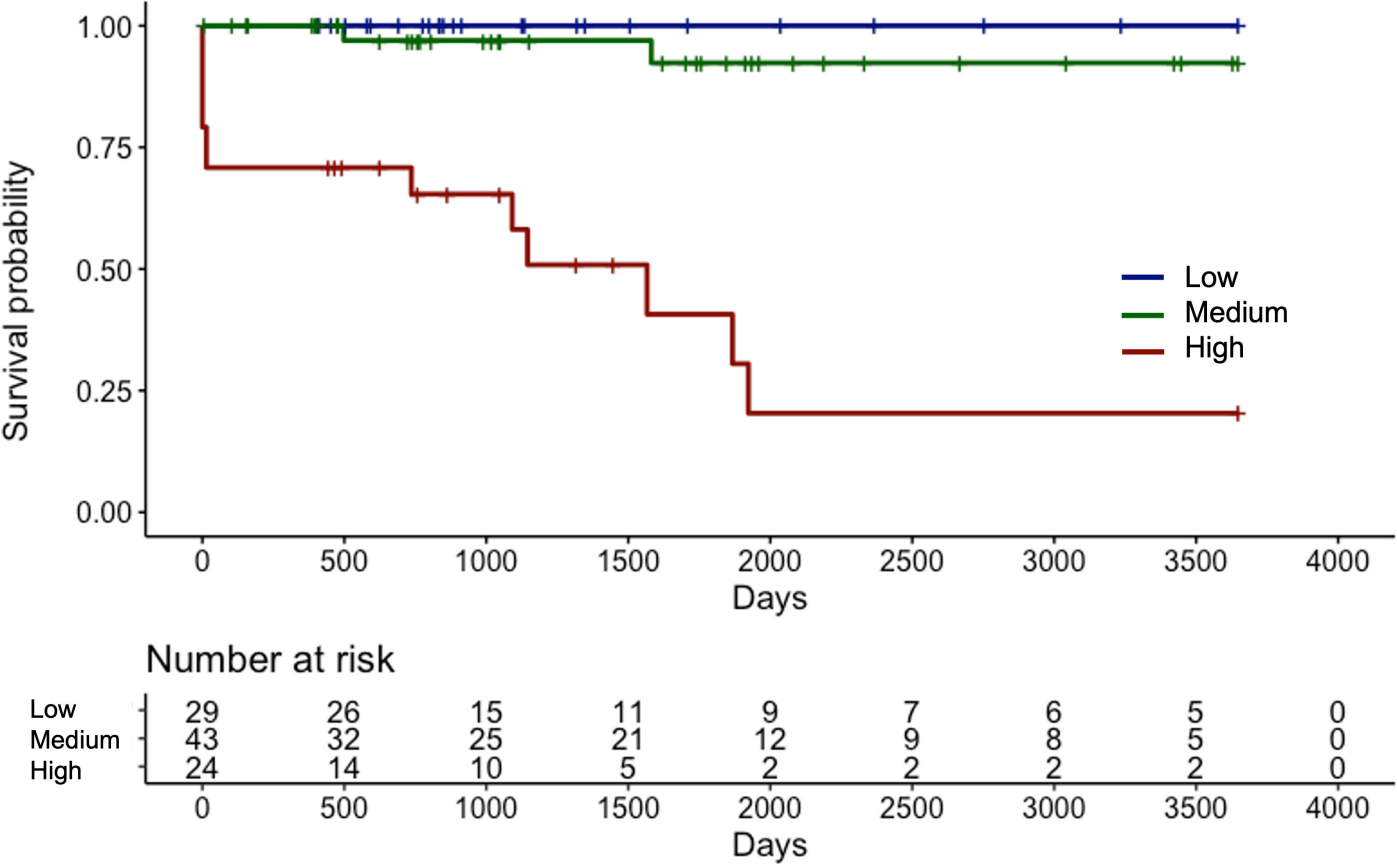
Kaplan-Meier curve demonstrating renal survival of Japanese patients with ANCA-associated renal vasculitis according to the renal risk score groups ANCA, anti-neutrophil cytoplasmic antibody.

**Figure 3:**
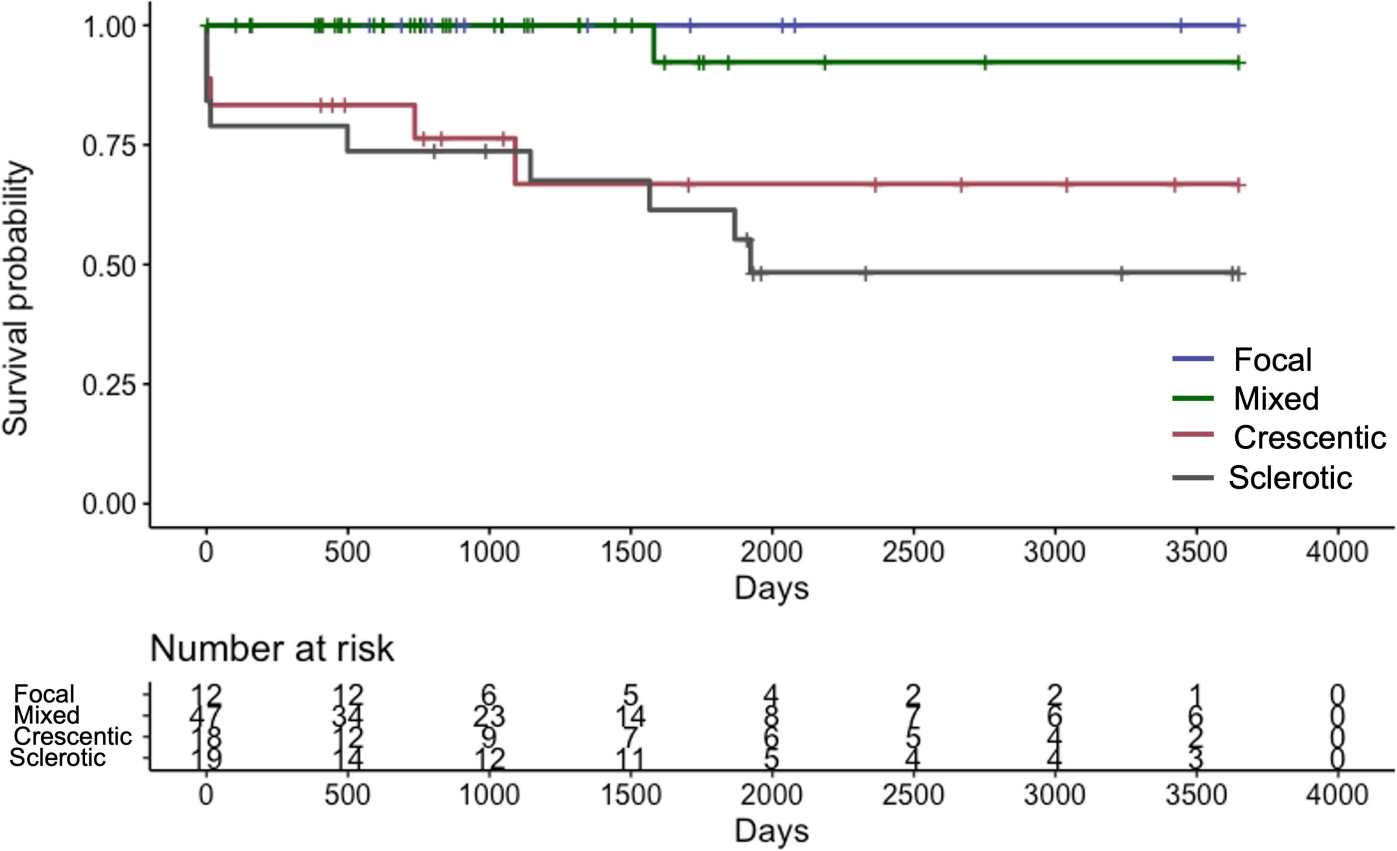
Kaplan-Meier curve demonstrating renal survival of Japanese patients with ANCA-associated renal vasculitis according to the histological classification ANCA: anti-neutrophil cytoplasmic antibody.

The median length of the observation period was 37.5 (IQR 21.5–77.0) months. Fifteen (15.6%) patients progressed to ESRD and required renal replacement therapy. Kaplan-Meier curve of RRS-predicted risk demonstrated that renal prognosis was the worst in the “high-risk” group and the best in the “low-risk” group (**Figure 2**). There were significant differences in renal survival rates among the three groups (*p<*0.001). In the histological classification, the Kaplan-Meier curve showed that renal survival rates deteriorated in the order of “focal” (best) to “mixed,” “crescentic,” and “sclerotic” (worst), respectively (**Figure 3**). There were significant differences in renal survival rates among the four groups (*p*<0.001); however, the order of the classes differed from that of Berden et al. (9).

### Risk factors related to ESRD

To identify risk factors associated with ESRD, we analyzed the baseline characteristics of the patients and the RRS using univariate and multivariable Cox regression analysis (**Table 3**). Univariate analysis showed that CRP and RRS were associated with ESRD. The multivariable analysis demonstrated that RRS was independently associated with ESRD among these factors.

**Table 3:** Independent risk factors for end-stage renal disease in patients with ANCA-associated renal vasculitis.

Variables	Univariate analysis		Multivariable analysis	
	HR (95%CI)	P-value	HR (95%CI)	P-value
Sex, males/females	1.497 (0.539-4.152)	0.438		
Age, per 1-yr increase	0.982 (0.946-1.029)	0.421		
WBC, per 100/µL	0.998 (0.983-1.013)	0.881		
Hb, per 1 mg/dL	0.940 (0.712-1.219)	0.651		
CRP, per 1 mg/dL increase	0.898 (0.772-0.996)	0.040*	0.904 (0.774-1.016)	0.094
BVAS, per 1 unit increase	0.926 (0.818-1.034)	0.181		
renal risk score, per 1 unit increase	1.648 (1.366-2.087)	<.0001*	1.723 (1.387-2.302)	<.0001*
History of hypotensive drugs, yes/no	1.482 (0.518-4.241)	0.465		
Smoking history, yes/no	2.254 (0.659-7.709)	0.185		
Use of antidiabetic drugs, yes/no	1.485 (0.184-11.975)	0.724		
Use of methylprednisolone pulse, yes/no	1.225 (0.387-3.880)	0.726		
Dose of prednisolone, per 10 mg increase	1.121 (0.743-1.730)	0.600		
Plasmapheresis, yes/no	1.743 (0.491-6.182)	0.417		
Cyclophosphamide, yes/no	0.692 (0.156-3.078)	0.614		

*p < 0.05. BVAS, Birmingham Vasculitis Activity Score; CI, confidence interval; CRP, C-reactive protein; Hb, hemoglobin; HR, hazard ratio; WBC, white blood cell count

### Predictive values of RRS and histological classification

**Figure 4:**
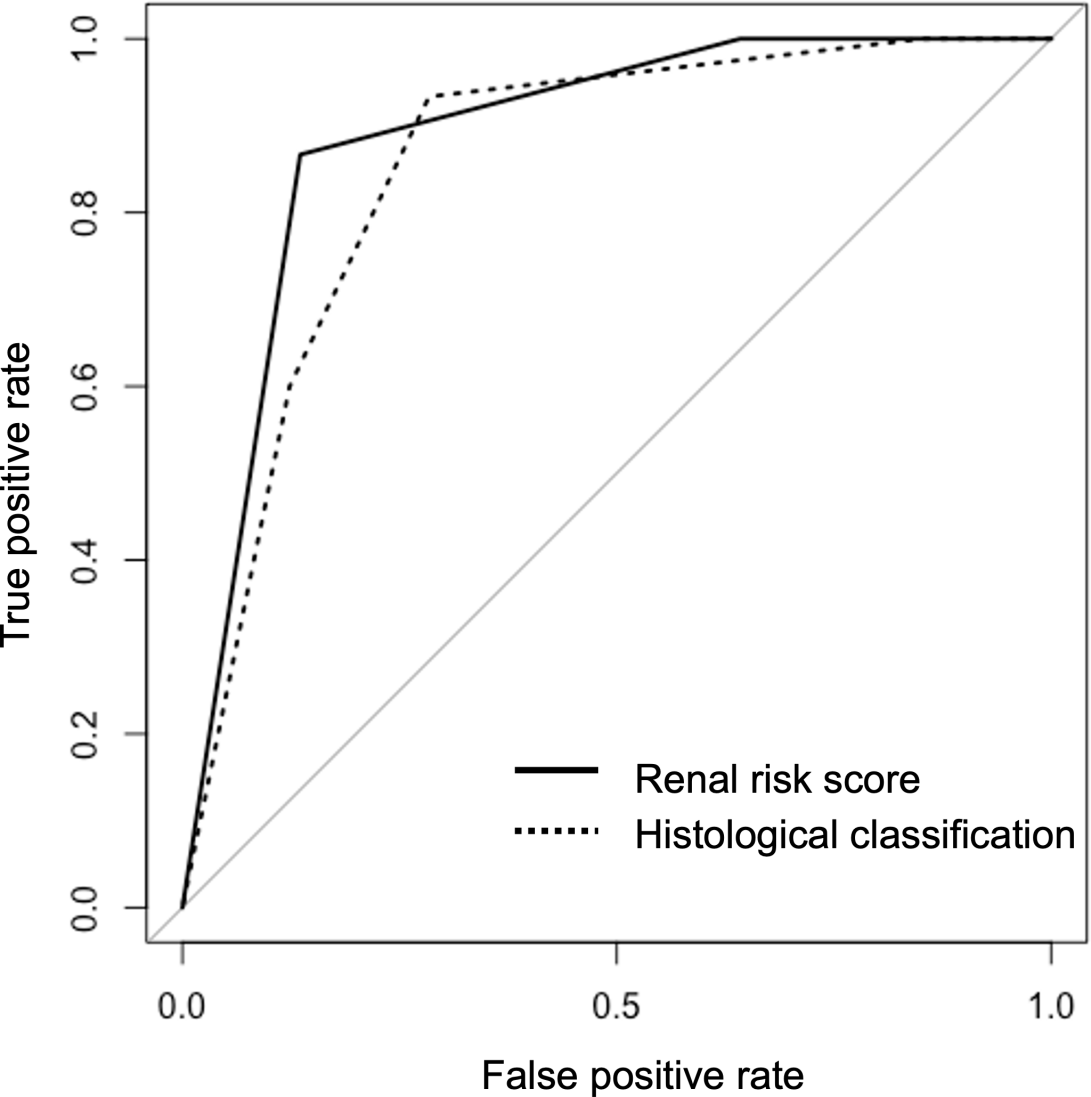
The receiver operating characteristic curves for predicting end-stage renal disease using the renal risk score and the histological classification.

The AUC value of RRS was 0.890 (95%CI, 0.819–0.959) for developing ESRD. The AUC value of the histological classification for progression to ESRD was 0.857 (95% CI, 0.773–0.942). Two receiver operating characteristic (ROC) curves are shown in **Figure 4** and did not show significant differences (p=0.474). We also calculated the AUC value using the points of RRS, which was 0.913 (95%CI, 0.833-0.994) for developing ESRD, and the cut-off value was 7.5 points (sensitivity: 86.7%, specificity: 86.6%) (**Supplementary Figure S1**).

## Discussion

Our present study demonstrated the usefulness of RRS for predicting renal survival among patients with AAGN in Japan. In 2010 Berden et al. devised a histological classification to predict renal outcomes in patients with AAGN (9). It categorized renal pathological findings into four groups: focal, crescentic, mixed, and sclerotic. Among their subjects, the focal group had the best renal survival, the crescentic class had the second-best, the sclerotic class had the worst renal prognosis, and the mixed class had the second-worst. However, several studies and meta-analyses have reported that the histological classification does not accurately predict the renal outcomes of the mixed and crescentic classes (10–13). Unlike the results of Berden et al. (9), our analysis found the mixed class had a better prognosis than that of the crescentic class. Several previous studies obtained similar results (24–26). We speculatively attributed these differences to the following considerations. First, in patients with AAGN in Japanese and Chinese patients, MPO-ANCA is more common than PR3-ANCA. Histological differences between MPO-AAV and PR3-AAV have been found in several reports (27–30): namely, more fibrotic changes, such as glomerulosclerosis, interstitial fibrosis, and tubular atrophy, are typically recognized in MPO-AAV than in PR3-AAV (28–30). Second, the rate of >25% normal glomeruli was lower in the crescentic class than in the mixed class (**Supplementary Table S2**). Hillhorst et al. showed that renal survival was significantly worse when the percentage of normal glomeruli was less than 25% (26).

Recently, Brix et al. proposed the RRS as another method to predict renal survival (14). We demonstrated that RRS was a stable predictor for ESRD in our analysis. In the baseline characteristics of our study, patient age was older, and MPO-ANCA positivity was higher than in the cohort of Brix et al. (14). Despite the differences in ANCA positivity between European and Asian cohorts mentioned above, the higher MPO-AAV rate did not degrade the ability of RRS to predict ESRD, not only in our cohort but in other Asian cohorts (19, 31). You et al. demonstrated that the prognostic value of RRS in Chinese patients with AAGN of the crescentic or mixed class was better than the histological classification (32). Saito et al. reported a validation study of RRS in Japanese patients with AAGN (19). They demonstrated that high-risk group patients had significantly poorer renal prognosis than other groups using the RRS, and that RRS was an independent renal prognostic factor in 86 Japanese biopsy-confirmed MPO-ANCA-positive GN patients. Our study likewise found the renal prognosis using RRS accurate in Japanese patients even when the histological classification was not predictive, and the rate of systemic AAV was higher than the report by Saito et al. (19).

In this study, we also evaluated how treatment for ANCA-associated renal vasculitis impacts the risk of ESRD. We found that the use of methylprednisolone pulse, dose of prednisolone, plasmapheresis, or cyclophosphamide did not significantly influence the risk of ESRD. Regarding the use of rituximab, only two patients were treated with it, and we did not include the analysis. We could confirm that RRS was the independent renal prognostic risk factor using multivariable Cox regression analyses, consistent with the previous report (19).

Next, we also analyzed the subgroup of patients with MPO-AAV and obtained the same results (data not shown).

We also compared the predictive value of RRS and histological classification using AUC values. In previous studies, An et al. compared the predictive values using ROC curves, and they showed that AUC values (95%CI) of RRS and histological classification were 0.742 (0.679–0.804) and 0.587 (0.515–0.659), respectively, with RRS having better performance (31). Saito et al. evaluated the AUC values (95%CI) of RRS and histological classification as 0.80 (0.69–0.91) and 0.74 (0.62–0.86), respectively (19). In our study, the AUC values of the RRS and the histological classification calculated based on the ROC for developing ESRD were 0.890 (95%CI, 0.819–0.959) and 0.857 (95% CI, 0.773–0.942), respectively. We found that the difference in prediction for renal survival between the RRS and the histological classification was insignificant. Assessment using the histological classification does not necessarily show a gradual deterioration in survival from “focal” (best) to “crescentic,” “mixed,” or “sclerotic” (worst), as was the case in our results. The evaluation results using the RRS were consistent across different cohorts, and we believe it is a highly reproducible prognostic tool.

There are several limitations to this study. First, this is a retrospective study, and the treatment regimen by clinicians changed over the years as new evidence came to light. Second, the number of patients and events did not provide robust evidence. Third, we must consider that the patients may have had a period of undiagnosed AAGN before the diagnosis was made. Fourth, there may be a selection bias for the patients with AAGN in this cohort since a bias would have been introduced by excluding cases where the patients refused biopsy. Fifth, there was a variation in the induction therapy protocol in our cohort over the course of a long-term follow-up period. More extensive and prospective studies are needed in the future.

## Conclusions

We demonstrated the stable predictive ability of the RRS for renal survival in Japanese patients over the long term, in contrast to the histological classification. However, further studies are necessary for validation.Abbreviations

AAV, anti-neutrophil cytoplasmic antibody-associated vasculitis; AAGN, anti-neutrophil cytoplasmic antibody-associated glomerulonephritis; ANCA, anti-neutrophil cytoplasmic antibody; AUC, area under the receiver operating characteristic curve; BVAS, Birmingham Vasculitis Activity Score; CRP, C-reactive protein; eGFR, estimated glomerular filtration rate; EGPA, eosinophilic granulomatosis polyangiitis; ESRD, end-stage renal disease; GPA, granulomatosis polyangiitis; IQR, interquartile range; MPA, microscopic polyangiitis; ROC, receiver operating characteristic; RRS, renal risk score.

## Data availability statement

The raw data supporting the conclusions of this article will be made available by the authors, without undue reservation.

## Ethics statement

The studies involving human participants were reviewed and approved by Institutional Review Boards of Nagasaki University. Written informed consent for participation was not required for this study in accordance with the national legislation and the institutional requirements.

## Author contributions

TU, KI: Conception and design of the study, analysis and interpretation of data, and drafting of the article. TU, SS: Statistical analysis and interpretation of data. TU, KI, AY, KM, MK: Collection and assembly of data. TU, KI, KM, MK, SS, NI, TN, AK: analysis, and interpretation of data, critical revision of the manuscript. TN, AK, supervision of the project. All authors contributed to the article and approved the submitted version.
