# Supplementary figures and images for: Evaluation of a renal risk score for Japanese patients with ANCA-associated glomerulonephritis in a multi-center cohort study
Source: Front Immunol. 2023 Feb 28;14:1141407. doi: 10.3389/fimmu.2023.1141407 (PMC10011144; doi:10.3389/fimmu.2023.1141407)

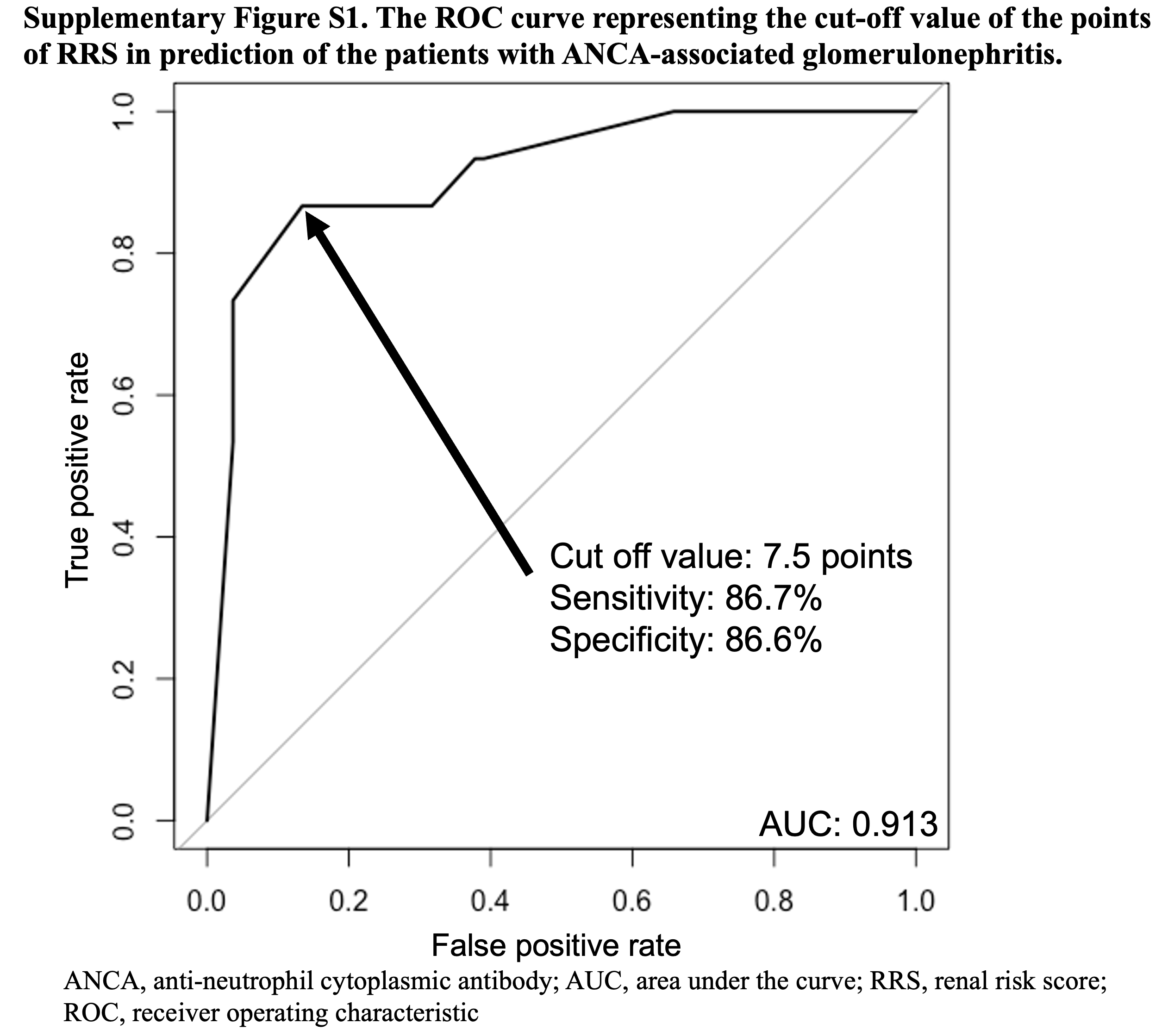

Supplement: Supplementary file 1 [file Image_1.tiff]
