# Supplementary material for: Evaluation of a renal risk score for Japanese patients with ANCA-associated glomerulonephritis in a multi-center cohort study
Source: Front Immunol. 2023 Feb 28;14:1141407. doi: 10.3389/fimmu.2023.1141407 (PMC10011144; doi:10.3389/fimmu.2023.1141407)
Supplement: Supplementary file 2 [file Table_1.docx]

**Supplementary Table S1. Baseline characteristics of patients with ANCA-associated renal vasculitis stratified by progression to ESRD or not.**

| **Variables** | Not progression to ESRD (n=81) | Progression to ESRD (n=15) |
| --- | --- | --- |
| **Male (n, %)** | 38 (46.9%) | 8 (53.3%) |
| **Age, yrs^†^** | 70 (63-77) | 68 (55-72) |
| **Diagnosis (n, %)** | MPA 68 (84.0%), GPA 10 (12.4%), RLV 3 (3.7%) | MPA 15 (100%), GPA 0 (0%), RLV 0 (0%) |
| **WBC (/μL) ^†^** | 8500 (6600-11400) | 8200 (6500-11600) |
| **Hb (g/dL) ^†^** | 9.8 (8.6-11.2) | 9.8 (8.8-10.6) |
| **CRP (mg/dL) ^†^** | 3.4 (0.3-10.0) | 1.5 (0.2-3.3) |
| **eGFR (mL/min/1.73㎡) ^†^** | 29.9 (19.4-43.1) | 8.3 (5.6-17.9) |
| **Proteinuria (n, %)** | 80 (98.8%) | 15 (100%) |
| **Hematuria (n, %)** | 81 (100%) | 15 (100%) |
| **Renal risk score^†^** | 2 (0-6) | 11 (8-11) |
| **MPO-ANCA positivity (n, %)** | 77 (95.1%) | 15 (100%) |
| **PR3-ANCA positivity (n, %)** | 5 (6.2%) | 1 (6.7%) |
| **BVAS^†^** | 14 (12-18) | 12 (12-16) |
| **Use of hypotensive drugs (n, %)** | 30 (41.1%) n/a 8 patient | 7 (50.0%) n/a 1 patients |
| **Smoking history (n, %)** | 28 (40.6%) n/a 12 patients | 7 (63.6%) n/a 4 patients |
| **Use of diabetes mellitus (n, %)** | 7 (9.3%) n/a 6 patients | 1 (7.1%) n/a 1 patients |
| **Use of methylprednisolone pulse (n, %)** | 50 (61.7%) | 11 (73.3%) |
| **Use of glucocorticoid (n, %)** | 79 (97.5%) | 15 (100%) |
| **Glucocorticoid dose (mg/day) ^†^** | 40 (30-50) | 40 (30-50) |
| **Plasmapheresis (n, %)** | 10 (12.4%) | 3 (20.0%) |
| **Cyclophosphamide (n, %)** | 17 (21.0%) | 2 (13.3%) |
| **Rituximab (n, %)** | 2 (2.5%) | 0 |
| **ESRD (n, %)** | 0 | 2 (4.7%) |

ANCA, anti-neutrophil cytoplasmic antibody; BVAS, Birmingham Vasculitis Activity Score; CRP, C-reactive protein; eGFR, estimated glomerular filtration rate; ESRD, end-stage renal disease; GPA, granulomatosis with polyangiitis; IQR, interquartile range; MPA, microscopic polyangiitis; MPO, myeloperoxidase; n/a, not available; PR3, proteinase-3; RLV, renal-limited vasculitis; WBC, white blood cell count

^†^Values are the median with IQR
