# Supplementary material for: Evaluation of a renal risk score for Japanese patients with ANCA-associated glomerulonephritis in a multi-center cohort study
Source: Front Immunol. 2023 Feb 28;14:1141407. doi: 10.3389/fimmu.2023.1141407 (PMC10011144; doi:10.3389/fimmu.2023.1141407)
Supplement: Supplementary file 3 [file Table_2.docx]

**Supplementary Table S2. Baseline characteristics of patients with ANCA-associated renal vasculitis categorized by the histological classification**

| **Variables** | Focal (n=12) | Crescentic (n=18) | Mixed (n=48) | Sclerotic (n=18) |
| --- | --- | --- | --- | --- |
| **Male (n, %)** | 6 (50%) | 8 (44.4%) | 24 (50%) | 10 (55.6%) |
| **Age, yrs^†^** | 67 (60.8-78.5) | 70 (64.8-75.3) | 72 (63.8-77) | 64.5 (53-73.5) |
| **Diagnosis (n, %)** | MPA 10 (83.3%) GPA 1 (8.3%) RLV 1 (8.3%) | MPA 16 (88.9%) GPA 1 (5.6%) RLV 1 (5.6%) | MPA 41 (85.4%) GPA 7 (14.6%) RLV 0 (0%) | MPA 16 (88.9%) GPA 1 (5.6%) RLV 1 (5.6%) |
| **WBC (/μL) ^†^** | 7435 (4600-12275) | 9500 (6375-10750) | 8750 (7125-11625) | 7630 (6550-11700) |
| **Hb (g/dL) ^†^** | 10.5 (9.0-12.8) | 9.8 (8.5-10.6) | 9.7 (8.4-10.9) | 9.8 (9.2-11.4) |
| **CRP (mg/dL) ^†^** | 6.17 (0.048-14.03) | 2.64 (0.11-9.59) | 2.91 (0.59-8.86) | 1.28 (0.21-5.30) |
| **eGFR (mL/min/1.73㎡) ^†^** | 58.6 (33.9-70.4) | 23.0 (8.7-30.2) | 32.1 (19.2-41.0) | 15.6 (8.1-24.4) |
| **Proteinuria (n, %)** | 12 (100%) | 18 (100%) | 47 (97.9%) | 18 (100%) |
| **Hematuria (n, %)** | 12 (100%) | 18 (100%) | 48 (100%) | 18 (100%) |
| **Percentage of normal glomeruli (n, %)** |  |  |  |  |
| **10-25%** | 0 | 3 (16.7%) | 7 (14.6%) | 3 (16.7%) |
| **<10%** | 0 | 9 (50.0%) | 7 (14.6%) | 10 (55.6%) |
| **>25% of TA/IF (n, %)** | 2 (16.7%) | 11 (61.1%) | 28 (58.3%) | 14 (77.8%) |
| **MPO-ANCA positivity (n, %)** | 12 (100%) | 18 (100%) | 45 (93.8%) | 17 (94.4%) |
| **PR3-ANCA positivity (n, %)** | 1 (8.3%) | 0 (0%) | 3 (6.3%) | 2 (11.1%) |
| **BVAS^†^** | 15 (14-18.8) | 14.5 (12-18.3) | 13.5 (10.5-18) | 12 (12-15.25) |
| **Use of hypotensive drugs (n, %)** | 4 (33.3%) | 5 (31.3%) n/a 2 patients | 20 (44.4%) n/a 3 patients | 8 (57.1%) n/a 4 patients |
| **Smoking history (n, %)** | 6 (50%) | 5 (33.3%) n/a 3 patients | 19 (44.2%) n/a 5 patients | 5 (50%) n/a 8 patients |
| **Use of diabetes mellitus (n, %)** | 1 (8.3%) | 1 (6.3%) n/a 2 patients | 5 (10.6%) n/a 1 patient | 1 (7.1%) n/a 4 patients |
| **Use of methylprednisolone pulse (n, %)** | 5 (41.7%) | 14 (77.8%) | 30 (62.5%) | 12 (66.7%) |
| **Use of glucocorticoid (n, %)** | 11 (91.7%) | 18 (100%) | 48 (100%) | 17 (94.4%) |
| **Glucocorticoid dose(mg/day) ^†^** | 40 (30-50) | 40 (40-42.5) | 40 (31.3-50) | 40 (30-46.3) |
| **Plasmapheresis (n, %)** | 0 | 5 (27.8%) | 7 (14.6%) | 1 (5.6%) |
| **Cyclophosphamide (n, %)** | 2 (16.7%) | 5 (27.8%) | 10 (20.8%) | 2 (11.1%) |
| **Rituximab (n, %)** | 0 | 1 (5.6%) | 1 (2.1%) | 0 |
| **ESRD (n, %)** | 0 | 5 (27.8%) | 1 (2.1%) | 9 (50%) |

ANCA, anti-neutrophil cytoplasmic antibody; BVAS, Birmingham Vasculitis Activity Score; CRP, C-reactive protein; eGFR, estimated glomerular filtration rate; ESRD, end-stage renal disease; GPA, granulomatosis with polyangiitis; IQR, interquartile range; MPA, microscopic polyangiitis; MPO, myeloperoxidase; n/a, not available; PR3, proteinase-3; RLV, renal-limited vasculitis; WBC, white blood cell count

^†^Values are the median with IQR
