# Supplementary material for: Evaluation of a renal risk score for Japanese patients with ANCA-associated glomerulonephritis in a multi-center cohort study
Source: Front Immunol. 2023 Feb 28;14:1141407. doi: 10.3389/fimmu.2023.1141407 (PMC10011144; doi:10.3389/fimmu.2023.1141407)
Supplement: Supplementary file 4 [file Table_3.docx]

**Supplementary Table S3. The baseline characteristics of the patients with ANCA-associated renal vasculitis categorized by the renal risk score**

| **Variables** | Low (n=29) | Medium(n=43) | High (n=24) |
| --- | --- | --- | --- |
| **Male (n, %)** | 18 (62.1%) | 15 (34.9%) | 13 (54.2%) |
| **Age, yrs^†^** | 67 (61-75.5) | 71 (65-78) | 68.5 (61.3-75.8) |
| **Diagnosis (n, %)** | MPA 26 (89.7%), GPA 2 (6.9%), RLV 1 (3.5%) | MPA 34 (79.1%), GPA 8 (18.6%), RLV 1 (2.3%) | MPA 23 (95.8%), GPA 0, RLV 1 (4.2%) |
| **WBC (/μL) ^†^** | 9300 (6700-12050) | 8000 (6900-11400) | 8050 (6250-10495) |
| **Hb (g/dL) ^†^** | 10.5 (9.7-12.6) | 9.4 (8.4-10.9) | 9.8 (8.6-10.3) |
| **CRP (mg/dL) ^†^** | 6.1 (0.9-13.5) | 2.6 (0.3-9.9) | 1.7 (0.2-3.7) |
| **eGFR (mL/min/1.73㎡) ^†^** | 45.1 (35.9-64.6) | 24.7 (16.8-33.0) | 11.5 (6.5-22.3) |
| **Proteinuria (n, %)** | 28 (96.6%) | 43 (100%) | 24 (100%) |
| **Hematuria (n, %)** | 29 (100%) | 43 (100%) | 24 (100%) |
| **Renal risk score^†^** | 0 (0-0) | 2 (2-5) | 9 (8-11) |
| **MPO-ANCA positivity (n, %)** | 29 (100%) | 40 (93.0%) | 23 (95.8%) |
| **PR3-ANCA positivity (n, %)** | 1 (3.5%) | 4 (9.3%) | 1 (4.2%) |
| **BVAS^†^** | 14 (12-18) | 14 (12-20) | 12 (12-14.8) |
| **Use of hypotensive drugs (n, %)** | 12 (42.9%) n/a 1 patient | 16 (42.1%) n/a 5 patients | 9 (42.9%) n/a 3 patients |
| **Smoking history (n, %)** | 15 (57.7%) n/a 3 patients | 10 (27.0%) n/a 6 patients | 10 (58.8%) n/a 7 patients |
| **Use of diabetes mellitus (n, %)** | 3 (10.3%) | 2 (5.1%) n/a 4 patients | 3 (14.3%) n/a 3 patients |
| **Use of methylprednisolone pulse (n, %)** | 15 (51.7%) | 28 (65.1%) | 18 (75.0%) |
| **Use of glucocorticoid (n, %)** | 29 (100%) | 41 (95.4%) | 24 (100%) |
| **Glucocorticoid dose (mg/day) ^†^** | 40 (32.5-50) | 40 (30-40) | 40 (36.3-47.5) |
| **Plasmapheresis (n, %)** | 0 | 10 (23.3%) | 3 (12.5%) |
| **Cyclophosphamide (n, %)** | 7 (24.1%) | 10 (23.3%) | 2 (8.3%) |
| **Rituximab (n, %)** | 1 (3.5%) | 1 (2.3%) | 0 |
| **ESRD (n, %)** | 0 | 2 (4.7%) | 13 (54.2%) |

ANCA, anti-neutrophil cytoplasmic antibody; BVAS, Birmingham Vasculitis Activity Score; CRP, C-reactive protein; eGFR, estimated glomerular filtration rate; ESRD, end-stage renal disease; GPA, granulomatosis with polyangiitis; IQR, interquartile range; MPA, microscopic polyangiitis; MPO, myeloperoxidase; n/a, not available; PR3, proteinase-3; RLV, renal-limited vasculitis; WBC, white blood cell count

^†^Values are the median with IQR
